# The BDNF Val^66^Met Polymorphism Influences Reading Ability and Patterns of Neural Activation in Children

**DOI:** 10.1371/journal.pone.0157449

**Published:** 2016-08-23

**Authors:** Kaja K. Jasińska, Peter J. Molfese, Sergey A. Kornilov, W. Einar Mencl, Stephen J. Frost, Maria Lee, Kenneth R. Pugh, Elena L. Grigorenko, Nicole Landi

**Affiliations:** 1 Haskins Laboratories, New Haven, CT, United States of America; 2 University of Connecticut, Storrs, CT, United States of America; 3 Yale University, New Haven, CT, United States of America; 4 University of Houston, Houston, TX, United States of America; 5 Baylor College of Medicine, Houston, TX, United States of America; 6 Moscow State University, Moscow, Russian Federation; 7 Saint-Petersburg State University, Saint-Petersburg, Russian Federation; 8 Moscow City University for Psychology and Education, Moscow, Russian Federation; Hangzhou Normal University, CHINA

## Abstract

Understanding how genes impact the brain’s functional activation for learning and cognition during development remains limited. We asked whether a common genetic variant in the *BDNF* gene (the Val^66^Met polymorphism) modulates neural activation in the young brain during a critical period for the emergence and maturation of the neural circuitry for reading. In animal models, the *bdnf* variation has been shown to be associated with the structure and function of the developing brain and in humans it has been associated with multiple aspects of cognition, particularly memory, which are relevant for the development of skilled reading. Yet, little is known about the impact of the Val^66^Met polymorphism on functional brain activation in development, either in animal models or in humans. Here, we examined whether the BDNF Val^66^Met polymorphism (dbSNP rs6265) is associated with children’s (age 6–10) neural activation patterns during a reading task (n = 81) using functional magnetic resonance imaging (fMRI), genotyping, and standardized behavioral assessments of cognitive and reading development. Children homozygous for the Val allele at the SNP rs6265 of the *BDNF* gene outperformed Met allele carriers on reading comprehension and phonological memory, tasks that have a strong memory component. Consistent with these behavioral findings, Met allele carriers showed greater activation in reading–related brain regions including the fusiform gyrus, the left inferior frontal gyrus and left superior temporal gyrus as well as greater activation in the hippocampus during a word and pseudoword reading task. Increased engagement of memory and spoken language regions for Met allele carriers relative to Val/Val homozygotes during reading suggests that Met carriers have to exert greater effort required to retrieve phonological codes.

## Introduction

Human development is characterized by a remarkable capacity to learn, and among the most complex challenges in childhood is learning to read proficiently. Our ability to learn to read results from the experientially- and biologically-guided maturation and organization of the brain. Although the brain continues to change throughout the lifespan, it undergoes greater organization in early life [1]. These developmental processes are partially under genetic control, driven by molecular signals that result in changes ranging from subtle tuning of synaptic connections to more large-scale functional organization of cortical areas that underlie human cognition [2]. Yet our knowledge of how genes impact the brain’s capacity for learning and cognition during development remains limited. Here we investigate how variation in the Brain Derived Neurotrophic Factor (*BDNF*) gene, which has an established role in brain maturation and plasticity, particularly as it pertains to cognition and memory, may contribute to variation in reading and related skills [3–8]. Specifically, we ask whether a common genetic variant in *BDNF*, the Val^66^Met polymorphism, alters patterns of neural activation in the developing brain in ways that are important for children’s cognitive development, and consequently their reading and other developing academic abilities.

### BDNF and Brain Development

The *BDNF* gene is located on chromosome 11p13 of the human genome and encodes brain derived neurotrophic factor (BDNF), which is a highly expressed growth factor governing the development and maturation of the central as well as the peripheral nervous systems. A common single nucleotide polymorphism (SNP) in the *BDNF* gene, Val^66^Met (dbSNP: rs6265) results in an amino acid substitution (valine to methionine) in proBDNF peptide [a precursor peptide to BDNF [9, 10]], at codon 66. The rs6265 variant is a missense (i.e., leading to the alteration of the amino acid composition of the protein) SNP with two alternative alleles—G (ancestral) and A (derived). Thus, three genotypes are possible for this SNP: GG, AG, and AA, corresponding to Val/Val, Val/Met or Met/Met, respectively.

This polymorphism has been extensively studied [11], and has been shown to affect secretion of BDNF [12] and regulate neuronal survival, morphology and function [13]. BDNF protein is a neurotrophin that influences many neural events related to brain plasticity [14] by regulating cell survival, proliferation and synaptic growth, and by modulating synaptic changes, particularly long-term potentiation (LTP) in the hippocampus. Individuals homozygous for the ancestral G allele (i.e., Val/Val homozygotes), when compared to heterozygous individuals (i.e., Val/Met) or individuals homozygous for the derived A allele (i.e., Met/Met), generally perform better in a variety of domains of cognition, including memory [15–21] attention [22–26], and executive function [3, 8, 27, 28], although this pattern of results is not always consistent in studies of *BDNF* and cognition (see Mandelman and Grigorenko [11] for a review).

Specifically, research with animal models has demonstrated that mice with higher brain BDNF levels showed enhanced spatial learning and memory function on a Morris Water Maze test [29], and mice with decreased BDNF levels in the frontal cortex showed impaired spatial working memory [30]. Human adults who are carriers of the Met allele at the Val^66^Met polymorphism have shown poorer performance on test measuring executive function and working memory, and corresponding reduced hippocampal volume [15], poorer performance on a recognition memory task [31], and greater activation in the hippocampus during an N-back memory task [12]. This literature suggests a relationship between variation in *BDNF* and individual differences in memory processes, which involve hippocampal and cortical structures. However, to date, human studies of genetic polymorphisms in *BDNF* have largely focused on adult cognition and brain function. Thus, there is both a theoretical and practical need to understand how variation in *BDNF* relates to complex cognitive function, in the developing brain.

*BDNF* is predominantly expressed in the postnatal brain, and peaks at a time in development most correlated with neuronal genesis and migration, differentiation, and synaptogenesis [2, 32–37]. However, *BDNF* is variably expressed over early life during periods critical for language and cognitive development and the expression of *BDNF* differs by brain regions. BDNF is associated with structural changes brain-wide, including hippocampal structure [38, 39], white matter [40, 41], and cortical and subcortical regions, e.g., cortical thickness and volume [39, 42–44]. Peak *BDNF* expression in the temporal cortex occurs in infancy, and decreases with age, whereas peak *BDNF* expression in the frontal cortex occurs in young adulthood, and *BDNF* expression in the hippocampus remains relatively constant over the lifespan [45]. Differential *BDNF* expression over cortical regions matches structural imaging evidence that different brain structures mature at different rates. Frontal brain regions develop more slowly than other regions, for example, gray to white matter ratio [46–49], synaptic density [50], and myelination [51–53] have protracted developmental trajectories in the frontal cortex which parallel those of children’s cognitive and academic abilities.

Children’s cognitive and linguistic abilities continue to develop concurrently through the early grade-school years, guided by the maturation of neural sites and systems that support them as they are learning how to read [54–56]. For example, developmental changes in the superior parietal lobule, a region known to support working memory, are associated with children’s working memory capacity as measured by a digit span task [57]. Children’s scores on measures of executive function over time were found to be related to voxel-based gray matter in frontal and temporal cortex, cingulate, insula, occipitotemporal regions (fusiform gyrus), and parietal regions (precuneus) [58].

Interacting “domain-general” cognitive processes including working memory and attentional selection support learning [59], including learning to read [60]. When reading, memory systems hold incoming information in an available state for further manipulation; short-term memory is predictive of reading decoding and fluency [61]. Working memory involves preserving incoming information while simultaneously processing this (and/or other) information [62]. Working memory consists of a central executive system that is responsible for processing and manipulating information, and visual-spatial and verbal subsystems [62]. Poor working memory ability, specifically in the verbal storage system, is associated with poor reading ability. Aspects of language including phonological awareness (the awareness of and ability to manipulate the sound units of language [63]), are critical components of skilled reading. The ability to attend to and maintain phonological units in a phonological memory loop is predictive of reading outcomes [64], for example, children with poor phonological memory show difficulties in later reading [65], and children with poorer growth in working memory ability are more likely to have reading disability [66]. Retrieving the meaning of words and comprehending passages involves multiple memory systems, short-term memory is involved in storing phonological codes, working memory is involved in maintaining information about words and their meanings as text is integrated to establish coherence and retrieve information from long-term memory [67, 68]. Working memory is a significant predictor of reading comprehension [69] and accounts for a significant proportion of variance in children’s reading comprehension ability [67]. However, the contribution of working memory to reading depends on the task, that is, specific relationships between working memory and orthographic, phonological, and naming processes, and sentence and passage comprehension are different [61, 68], and correlations between working memory and comprehension are different with the addition of secondary tasks (e.g., multitasking) [70].

Despite established links between “domain general” cognition, (specifically working memory), and reading, the biological underpinnings of these relationships are still unclear. The role of *BDNF* in brain maturation and cognition, and its varied expression patterns over developmental periods corresponding to general cognitive development, led us to ask whether genetic variations in the *BDNF* gene (here, specifically the Val^66^Met polymorphism) may modulate patterns of neural activation in the developing brain that support cognitive skills important for literacy. A number of “candidate genes” for reading disorders have been identified, for example dyslexia susceptibility 1 candidate 1 (*DYX1C1*), roundabout Drosophila homolog 1 (*ROBO1*), doublecortin domain-containing protein 2 (*DCDC2*) and *KIAA0319* [71–74]. However, there is wide phenotypic heterogeneity across samples from which relationships between specific candidate genes and reading ability have been observed [75]. Further, work by Plomin and colleagues suggests that so called “generalist genes” such as *COMT* and *BDNF* with known impacts on general cognitive function may contribute significantly to reading ability and reading (and other learning) disabilities [76–78]. *BDNF* specifically may have a role in skilled reading because the gene has known roles in brain maturation, learning, and cognition. Moreover, the *BDNF* gene 11p13 susceptibility alleles have been associated with language impairment [79]. The *BDNF* Val^66^Met polymorphism represents a common variant in the population, as such, it may account for a meaningful amount of the variability in reading and other cognitive abilities. In this study, we tested the hypothesis that the *BDNF* Val^66^Met polymorphism is important for functional development of neural circuits underlying the cognitive processes that support reading development. The developing circuitry for word reading has been extensively studied; initially visual information about a word is relayed to an occipitotemporal region referred to as the “visual word form area” (VWFA; [80, 81, 82]). After initial input, a large left hemisphere circuit that translates the visual form into phonological and semantic information is engaged, including; the supramarginal gyrus (BA 40), which is involved in converting orthography into phonology [83], and the superior temporal gyrus (STG, BA 21/22/42), which is known to be important in phonological processing (e.g., Petitto, Zatorre [84] and Zatorre and Belin [85]); the temporoparietal system which includes the inferior parietal lobule (IPL), with the angular gyrus (BA 39), which is involved in lexical-semantic processing (Seghier, Fagan, & Price, 2010); and the L. Inferior frontal gyrus (IFG) which is involved in both phonological and semantic processing, as well as working memory, which is particularly important for larger units of text [83, 86–88].

More recently, subcortical regions have also been found to play a role in neural circuitry for reading including the thalamus, basal ganglia and hippocampus [89, 90]. The hippocampus is involved in memory function, including long-term memory and working memory [91, 92]. Performance on working memory tasks can be disrupted by hippocampal damage [93]. Patients with lesions to the medial temporal lobe show dramatic deficits in long-term memory [94]; this evidence has been one of the clearest examples of evidence for memory function in the hippocampus. Neurodevelopmental changes in these brain regions support the development of cognitive abilities throughout childhood that are important for literacy acquisition [90, 95–100].

Here we examine the relationship between the *BDNF* Val^66^Met polymorphism, brain activation, and reading using functional magnetic resonance imaging (fMRI) in combination with behavioral indices of reading development. We examined patterns of neural activation as children read words and pseudowords while undergoing fMRI neuroimaging. This particular word and pseudoword reading task has previously been show to recruit the brain’s language and reading circuitry and discriminate good from poor readers [89, 101–103]. We compare words (which has associated meanings) to pseudowords, which have no associated meaning, but are orthographically and phonologically similar to words. Words and pseudowords similarly engage the brain’s reading circuit, but differ in the search and retrieval of meaning from the lexicon, and thus put different demands on the sematic memory system.

Our behavioral battery included a comprehensive assessment of language and reading ability, including children’s phonological awareness and phonological working memory, oral and reading comprehension ability, letter-sound identification, spelling, word and passage reading, recall of information from a story, well as IQ (see “assessment battery” below for specific test details). This assessment battery is specifically selected to test children’s abilities across multiple domains of skilled reading. To the best of our knowledge, no study has yet examined whether variation in *BDNF* (i.e. Val^66^Met polymorphism) has an impact on patterns of activation in the developing brain for reading and specific abilities that are required for skilled reading.

This combined “genes-brain-behavior” approach to developmental research can provide new insights into the biological underpinnings of a complex psychological phenotype, such as reading ability and its underlying componential skills such as working memory and phonological processing.

## Materials and Methods

### Participants

**Fig 1:**
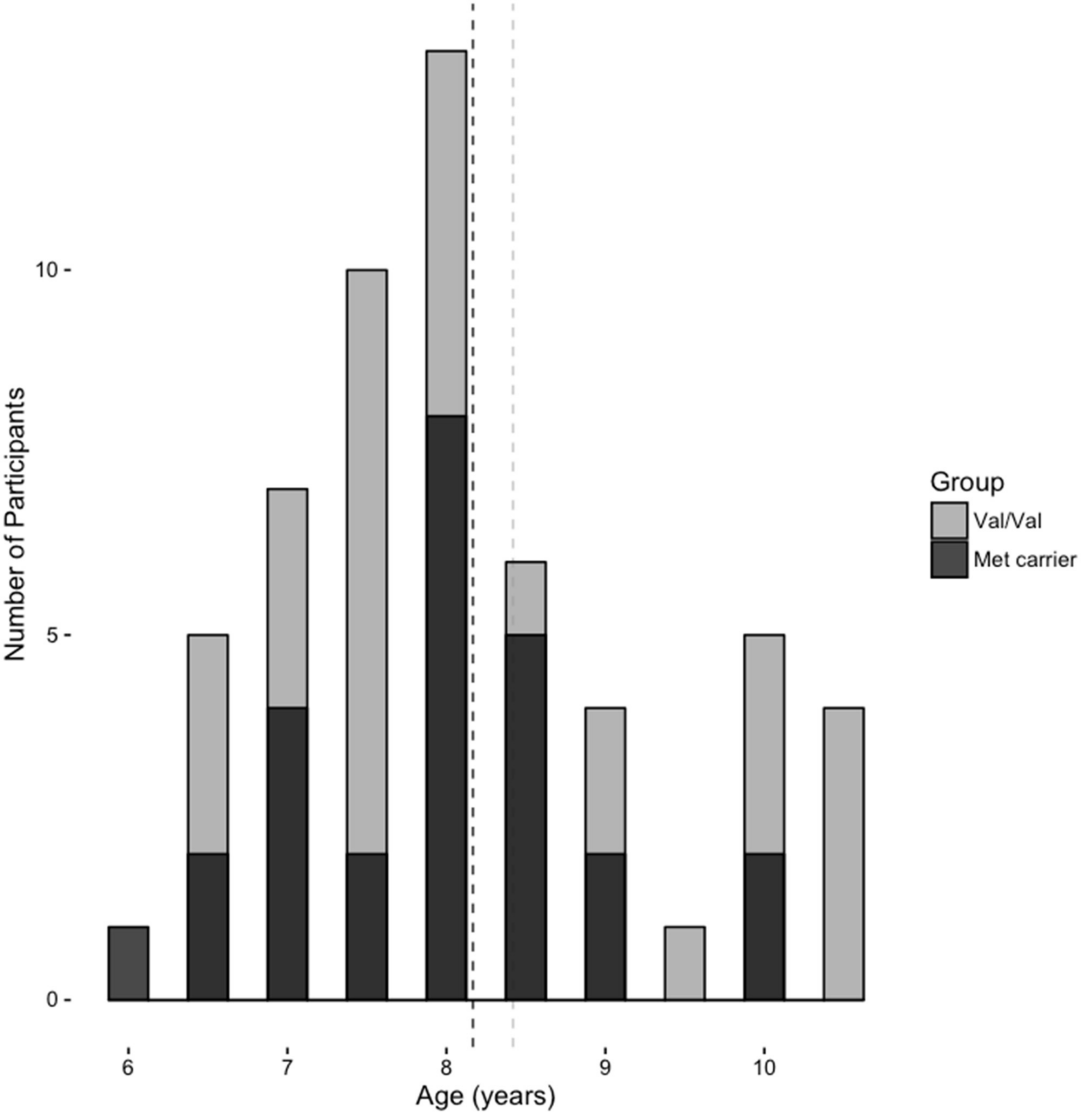
Participant Age. Histogram of participant ages by genotype group.

Eighty-one children between the ages of 6 and 10 (45 males, 36 females, mean age = 8.1, *SD* = 1.1) participated in this study (see Table 1 and Fig 1). The participants in this study are part of a larger longitudinal study investigating genetic links to structural and functional brain changes over a period in development corresponding to reading acquisition. Participants for this study were excluded if they had a standardized performance IQ below 80. This study also excluded children with a history of severe developmental or neuropsychological disorders. All children had normal or corrected to normal vision and normal hearing. All children had reading abilities within the typical range. From the larger longitudinal study sample, participants who had completed the behavioral battery, fMRI task, and had donated a saliva sample were included.

**Table 1:** Participant characteristics by genotype group.

	Val/Val	Met allele carriers	*p*
**n**	55	26	
**Age (years)**	8.2	7.9	0.546
**Gender (male:female)**	28:27	17:9	0.410
**Handedness (right:left)**	46:5	21:5	0.258

Participants were divided into two groups based on *BDNF Val* ^*66*^*Met* genotype: 1) Val/Val homozygotes (Val/Val; n = 55, 63%), and 2) Met allele carriers (n = 26, 37%) comprised primarily of Val/Met (n = 23, 28%) and, due to its low frequency, only a few Met/Met (n = 3, 9%). Thus we had two groups: the Val/Val homozygotes group and the Met allele carriers (combined Val/Met and Met/Met groups). The derived/minor allele frequency (MAF, here for the Met allele) was .23 (The distribution of alleles did not violate Hardy-Weinberg equilibrium, *p* = 0.7598). There were no significant differences between our two genotype groups in age, *F*(1,79) = 1.363, *p* = 0.248, grade, χ^2^ (6) = 5.588, *p* = 0.471, gender, χ^2^ (1) = 1.784, *p* = 0.410, or handedness, χ^2^ (1) = 3.379, *p* = 0.185. The two groups also did not differ with respect to word reading ability (average Woodcock Johnson Reading Ability; *F*(1,79) = 0.322, *p* = 0.572). Supplementary information about race and ethnicity, as well as history of stress can be found in the Appendix.

This study was approved by the Yale University Institutional Review Board. Parents of children participating in our study provided written consent and children provided verbal assent. Participant consent was recorded on a consent form that explained the details of the study, potential risks and benefits, and mechanism for storage of data and of identifying information.

### Behavioral Assessments

Participants completed a battery of cognitive, language and reading assessments as well as educational and neuropsychological history evaluations, including screening for ADHD. Several assessments from the Woodcock-Johnson Achievement Battery III [104] were administered, including letter-word decoding, pseudoword reading (“Word Attack”), spelling, oral language, story recall, passage comprehension, and oral comprehension. We also administered The Comprehensive Test of Phonological Processing (CTOPP; [105]), which includes measures of phonological awareness and phonological memory. Finally, participants also completed an IQ assessment using the Wechsler Abbreviated Scale of Intelligence, which include four subtest: Vocabulary, Verbal Similarities, Matrix Reasoning, Block Design (WASI; [106]). Parental reports of inattention and hyperactivity were collected from the SNAP-IV Parent Rating Scale [107, 108]. See S1 Table.

### fMRI Task

**Fig 2:**
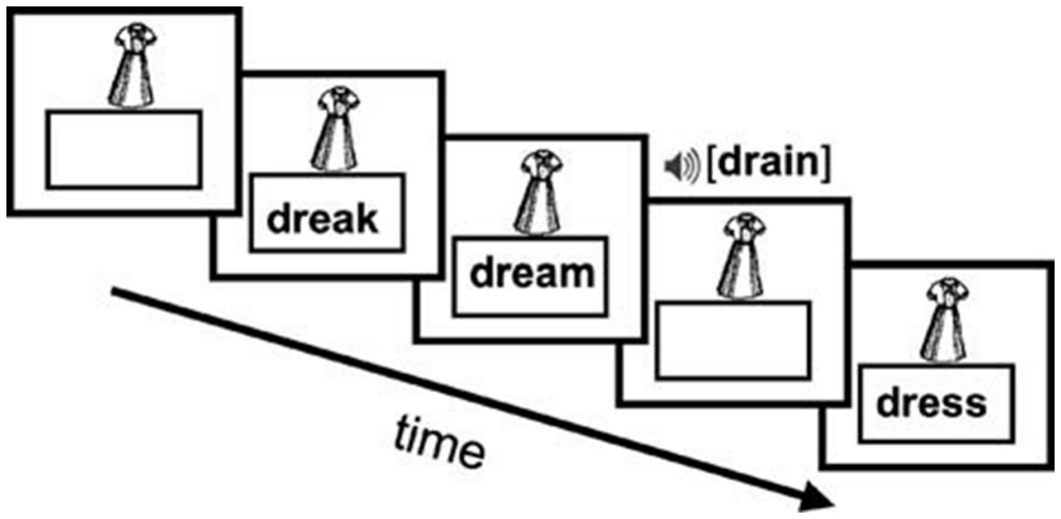
Schematic of fMRI paradigm. A picture cue is displayed and participants make identity match/mismatch judgments to print and speech tokens.

We used a cue-target identity task that required a match/mismatch judgment on each trial via a button press [101]. The task required participants to view pictures of common objects (e.g., a dress)—these pictures were followed by presentation of a single word or pronounceable, pseudoword (see Fig 2). For example, participants saw an image of a dress and then saw or heard the word ‘dress’ or a similar pseudoword ‘dreak’. Participants were asked to press one button when the picture and word matched (match condition) or press a different button when the picture and word did not match (mismatch condition). The use of an active task with a participant response allowed us to determine whether participants were reading accurately and attending to the stimuli. Real words were high frequency and 4–5 letters in length. Pseudowords were also 4–5 letters in length and phonotactically legal. Words and pseudowords were presented either visually or auditorily. Visual stimuli were presented for 2,000 ms and auditory stimuli were presented through an MRI-compatible headphone. Picture cues were treated as a trial condition and initially presented on the screen alone, allowing sufficient time to model separately the evoked responses to processing of the picture cues and for participants to encode the picture for comparison to the stimuli on subsequent trials. The majority of trials (80%) were mismatches, and only data from mismatch trials were included in analyses so that brain responses were compared on a common “mismatch” decision. Six types of mismatch trials were presented: spoken and printed high-frequency (HF) monosyllabic real words (e.g., DREAM); spoken and printed monosyllabic pseudowords (e.g., DREAK); printed HF monosyllabic words that are semantically related to the picture (e.g., SHIRT), and printed consonant strings (e.g., DRLST). Print stimuli were displayed in the box beneath the picture cue for 2,000 ms in 18-point Verdana font and speech stimuli were presented through MR compatible headphones. Our baseline was a rest periods during which children viewed a fixation cross. Stimulus presentation and response collection was controlled by a PC running E-prime 1.2 (Psychology Software Tools, Pittsburgh, PA, USA).

Reaction time and accuracy rates were recorded for each trial (32 trials per each condition in total). We compared groups (Val/Val homozygotes vs. Met allele carriers) on reaction time and accuracy rate using ANCOVA with age and parental reports of inattention & hyperactivity as covariates.

In the current analysis, we focused on only printed words and pseudowords to assess patterns of neural activation underlying specifically word reading, and not auditory word processing. This task is appropriate for children who are beginning readers and has been previously shown to discriminate good from poor readers [89, 101–103].

### Behavioral Data Analysis

We analyzed participants’ performance by genotype on our behavioral assessments using a MANCOVA. We assessed the effect of the genotype (Val/Val homozygotes versus Met allele carriers) on Letter-Word Decoding, Pseudoword Reading, Passage Comprehension, Oral Comprehension, Phonological Awareness, Phonological Memory and IQ, while treating age and parent reports of hyperactivity (from our ADHD screener) as covariates. The MANCOVA was followed with two post-hoc analyses: separate ANOVAs and linear discriminant analysis. We also analyzed correlations between all behavioral variables. All analyses were completed using R software [109].

### fMRI Data Processing and Analysis

Data were acquired using a Siemens Sonata 1.5-Tesla MRI Scanner. Image processing and statistical analyses were completed using the Analysis of Functional Neuroimages software package (AFNI; [110]). Twenty axial-oblique anatomic images were acquired, parallel to the intercommissural line based on sagittal localizer images. Activation images were acquired using a single-shot gradient echo, echo-planar pulse sequence at these twenty slice locations. Additional high-resolution anatomical images were collected for 3D co-registration. The imaging parameters for activation images were as follows: TE of 50 ms, TR of 2000 ms, flip angle of 80 degrees, FOV of 20 x 20 cm, matrix size of 64 x 64, slice thickness of 6 mm without spacing. A maximum of 10 imaging runs were collected for each participant. Images were corrected for slice acquisition time, motion-corrected, and transformed to standardized reference space defined by the Montreal Neurological Institute (MNI) by mapping the participant’s high-resolution anatomical scan to the ‘Colin27’ brain, using a combination of affine linear and non-linear registration parameters [111, 112]. Data were then spatially smoothed with a 6.25-mm FWHM Gaussian filter. Images were excluded if they exceeded an image-to-image change of 5 mm displacement in translation or a combination of rotation and shift exceeding a Euclidian Norm of 0.5. Regression-based estimation was used for the hemodynamic response at each voxel and each condition.

We implemented AFNI’s 3dREMLfit command for multiple regression, which adjusts for serial correlations in the time series noise, and therefore improves the accuracy of parameter and variance estimates. We used a single parameter gamma reference function with formula (t/(8.6*0.547))^8.6 * exp(8.6-t/0.547) to estimate the mean response for each condition and generate individual activation maps. We included six motion parameters that were obtained from motion correction step in preprocessing into our model as nuisance variables, as well as a Legendre polynomial set (from zero to third order) for each run to account for drift. Fixation periods comprised the baseline for the regression model and thus were not explicitly modeled with a regressor in our model.

We performed an ANCOVA group analysis using AFNI’s 3dMVM program [113]. We compared patterns of neural activation between our homozygous Val/Val vs. Met allele carriers for each condition (printed and words and pseudowords) with gender, age and IQ as covariates. Individuals’ voxel-wise response estimates (beta-weights) and their corresponding t-values for each stimulus type and/or contrast of interest were inputs to our group-level analysis in 3dMVM. We corrected for multiple comparisons using a cluster-wise threshold of .05, corresponding to a cluster size of 309. Cluster sizes were calculated using AFNI’s 3dClustSim program.

### Brain-Behavior Analysis

Using R [109], we computed partial correlations between behavioral scores on our test battery and activation in each brain region where significant group differences in activation were observed, while controlling for participant age. Nonparametric permutation testing (1000 permutations) was performed to estimate the significance of each correlation and adjust for multiple statistical tests. Random permutations were generated independently. For each permutation, the variables to be correlated were randomly exchanged, and then the statistical test was recalculated in each permutation. The p-value of each observed correlation was corrected by calculating the proportion of the 1000 permutations for which the generated correlation was greater than the observed correlation and then normalized by the number of permutations.

### DNA Collection and Analysis

During behavioral testing sessions with participants, we obtained biological samples using sterile Oragene^™^ saliva collection kits (DNA Genotek, Inc). DNA was extracted from the samples using the manufacturer’s protocol. We used the Applied Biosystems Inc. (ABI) TaqMan protocol for SNP genotyping. Specifically, the Assays-on-Demand^™^ SNP Genotyping Product containing forward and reverse primers as well as the probe for the SNP of interest was utilized. In order to amplify the region of interest, a polymerase chain reaction (PCR) was carried out using MJ Research Tetrad Thermocycler on a 384-well plate format. TaqMan reactions included 100 ng of genomic DNA, 2.5 μl of ABI Taqman^®^ Universal PCR Master Mix, 0.2 μl of ABI 40X Assays-on-Demand^™^ SNP Genotyping Assay Mix (assay ID C__11592758_10), 2.0 μl of sterile H2O and 0.5 μl of Bovine Serum Albumin (BSA). The genotyping call rate was 92%; quality was controlled by regenotyping.

## Results

### Behavioral

#### Standardized assessments

**Fig 3:**
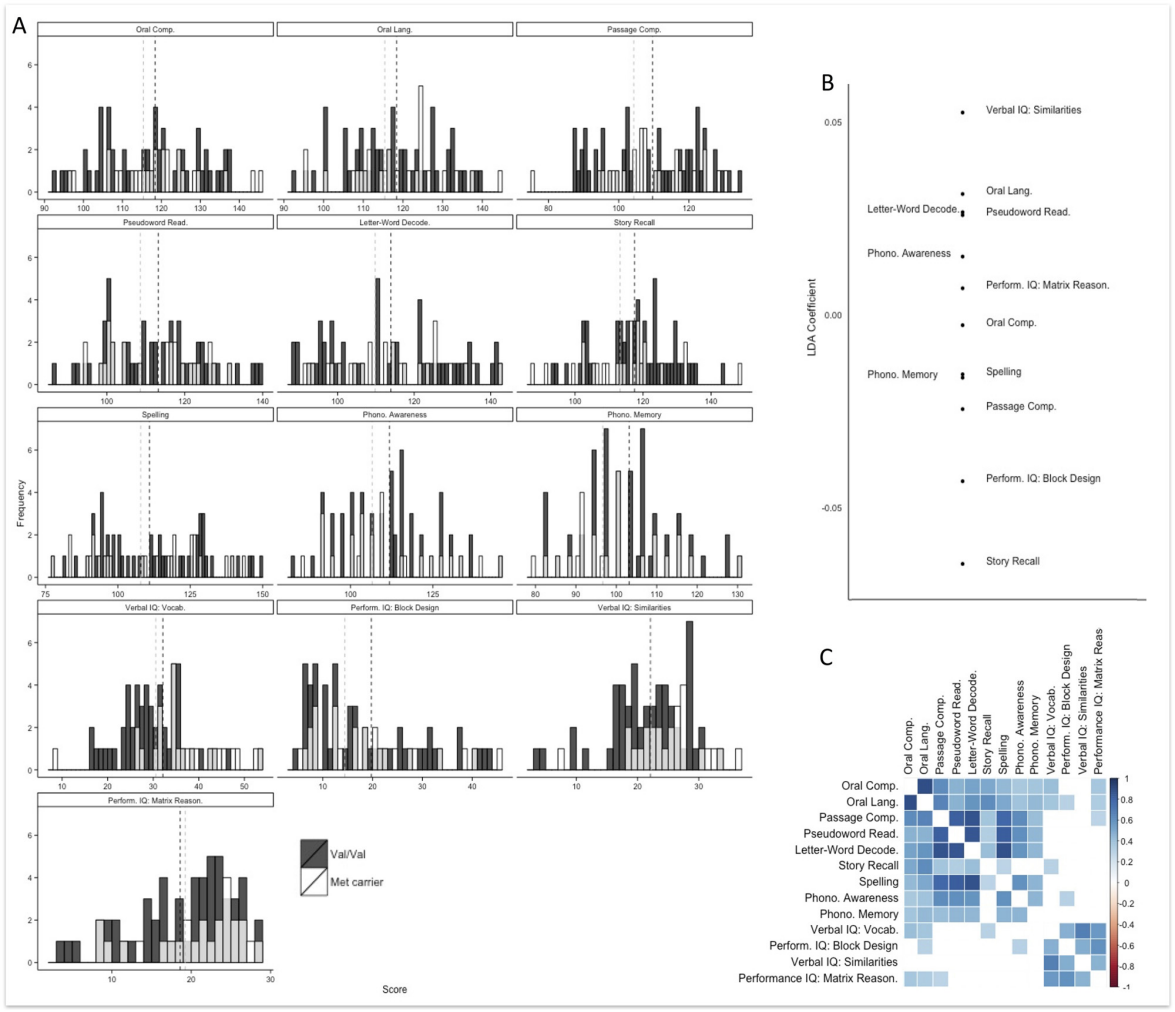
Behavioral Results. (A) Histograms of scores for each behavioral assessment, by group. Group means are indicated on each plot (B) Coefficients of linear discriminant function to classify individuals according to genotype group. Similar coefficients are shown for phonological memory, passage comprehension, block design, and story recall. (C) Significant correlations (p <.004) between behavioral assessments.

We found a significant main effect of genotype group, *F*(1,69) = 2.266, *p* = .017, 1-Wilk’s *λ* = .660, and age, *F*(1,69) = 13.4630, *p* < .001, 1-Wilk’s *λ* = .246. We did not observe a significant age by genotype group interaction in the MANCOVA. Table 2 shows the effect of the genotype group for each behavioral assessment. We also found significant positive correlations between our language and reading measures, and between IQ measures. All significant correlations are shown in Fig 3, *p*-values are Bonferroni corrected to *p* =.004.

**Table 2:** Behavioral Results.

Test	F	p	d	Group	Mean	SE
Letter-Word Decoding	1.514	0.223	0.272	Val/Val	113.915	2.037
				Met carrier	109.802	2.974
Nonsense Word Reading	3.22	0.077	0.391	Val/Val	113.193	1.577
				Met carrier	108.605	2.303
Spelling	0.487	0.488	0.169	Val/Val	110.9	2.409
				Met carrier	107.881	3.518
Passage Comprehension	4.289	0.042*	0.406	Val/Val	109.647	1.773
				Met carrier	104.292	2.589
Oral Comprehension	1.46	0.231	0.238	Val/Val	118.388	1.7
				Met carrier	115.382	2.477
Oral Language	1.481	0.223	0.249	Val/Val	118.403	1.612
				Met carrier	115.423	2.354
Story Recall	2.232	0.693	0.366	Val/Val	117.499	1.559
				Met carrier	113.255	2.275
Phonological Awareness	2.64	0.109	0.37	Val/Val	111.81	1.903
				Met carrier	106.584	2.778
Phonological Memory	5.65	0.020*	0.595	Val/Val	103.176	1.475
				Met carrier	96.658	2.154
Verbal IQ—Vocabulary	0.762	0.386	0.211	Val/Val	32.115	0.985
				Met carrier	30.554	1.473
Verbal IQ—Word Similarities	0.005	0.947	0.016	Val/Val	22.101	0.753
				Met carrier	22.194	1.127
Nonverbal IQ—Block Design	4.604	0.035*	0.509	Val/Val	19.742	1.391
				Met carrier	14.42	2.081
Nonverbal IQ—Matrix Reasoning	0.17	0.681	0.102	Val/Val	18.604	0.828
				Met carrier	19.238	1.238

F-values, p-values and effect sizes for the group comparison (MANCOVA) as well as means and standard errors for the Val/Val and Met allele carrier groups on our behavioral assessments.

* denotes p values less than 0.05.

Given the observation of a significant main effect of genotype, we followed this analysis with two post-hoc analyses, (1) separate univariate ANOVAs to evaluate which individual variables differ between groups and (2) a linear discriminant analysis (LDA) to evaluate which linear combination of variables best separates genotype groups. (1) Our ANOVAs revealed significantly better performance in Val/Val homozygotes relative to Met allele carriers on measures of Passage Comprehension (a component of the Woodcock Johnson Achievement Battery), Phonological Memory (a component of CTOPP phonological processing measure) and IQ (Block Design), but no significant differences between Val/Val homozygotes relative to Met allele carriers for Letter-Word Decoding, Pseudoword Word Reading (“Word Attack”), Oral Language, Story Recall, Spelling, Oral Comprehension (components of the Woodcock Johnson Achievement Battery), Phonological Awareness (a component of CTOPP phonological processing measure), or the remaining IQ subtests. These analyses also revealed significant effects of the age covariate on Spelling, Letter-Word Decoding, Pseudoword Reading, Phonological Memory, Verbal IQ Vocabulary and Verbal Similarities, Performance IQ Block Design and Matrix Reasoning. Mean scores and standard deviations for each behavioral assessment by group are shown in Table 2 and score distributions are show in Fig 3. (2) LDA revealed a discriminant function that classified individuals into genotype groups with 75% accuracy. The coefficients of the discriminant function were similar for tests with greater memory components (phonological memory, passage comprehension, block design, and story recall), and differed from tests that were predominantly verbal measures such as verbal IQ (vocabulary, word similarities) and oral language. This confirmed our ANOVA findings above. See Fig 3.

#### In-Scanner Matching Task Behavioral Response

No significant differences in reaction time and accuracy rates to making a picture-word matching judgment were observed between Val/Val homozygotes and Met allele carriers (Reaction Time: M_Val/Val_ = 1649 ms, SD = 354 ms; M_Met carrier_ = 1659 ms, SD = 342 ms, *F*(1,74) = 0.3263, *p* >.05; Accuracy: M_Val/Val_ = 86%, SD = 15%; M_Met carrier_ = 85%, SD = 18%, *F*(1,74) = 0.0143, *p*>.05). As such, differential patterns of functional activation in brain as a function of genotype cannot be due to simple performance differences.

### fMRI

We analyzed participants’ patterns of neural activation by the genotype (Val/Val homozygotes vs. Met allele carriers) and word type (word vs. pseudoword) while treating age, gender and parent reports of hyperactivity (from our ADHD screener) as covariates. There was a significant main effect of genotype and word type.

#### Word Type: Words vs. Pseudowords

**Fig 4:**
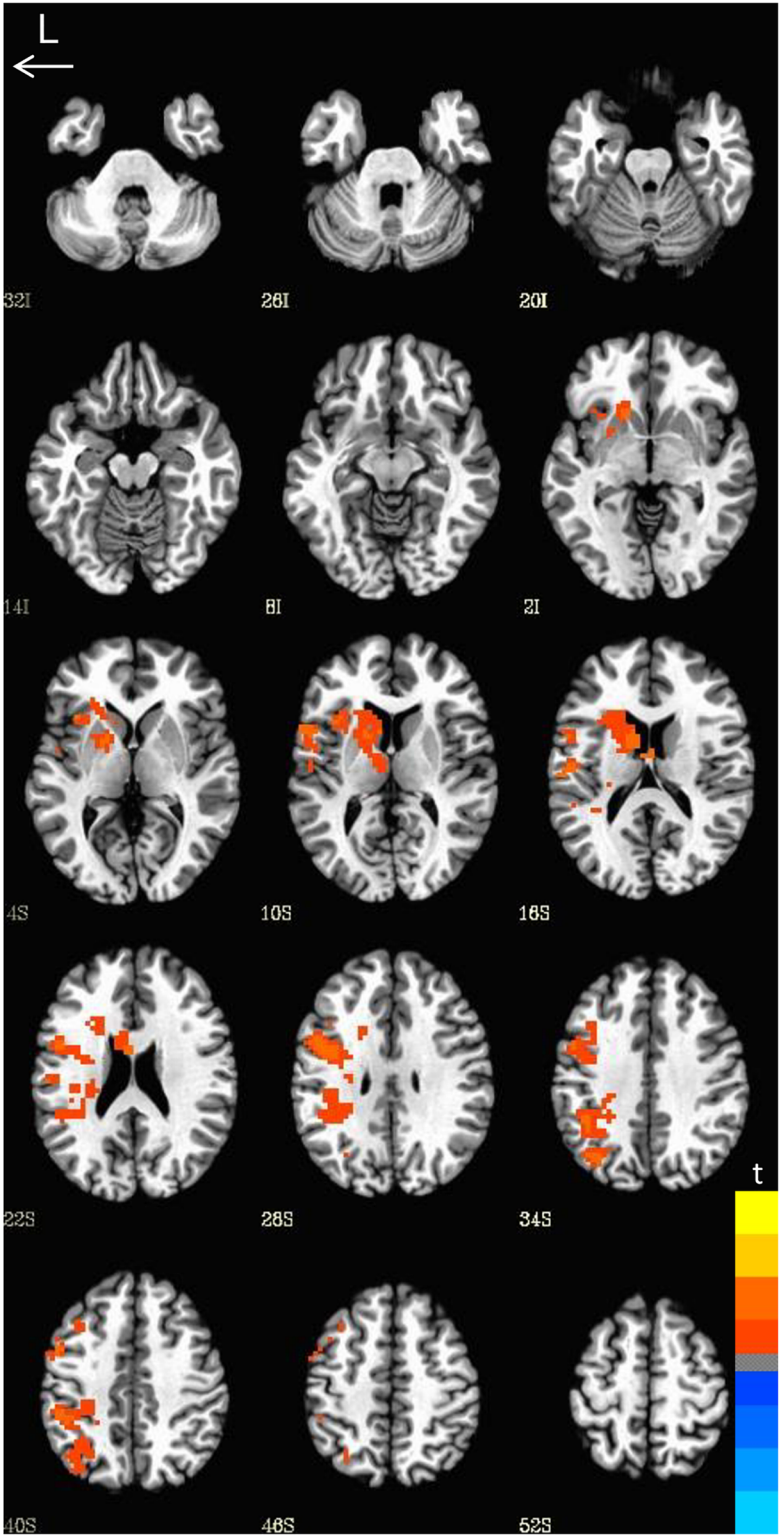
Neural activation by Condition. Comparison of neural activation during word versus pseudoword reading. Greater activation was observed for pseudowords relative to words.

We observed a significant main effect of word type (*t* = 21.993, *p* =.05; FWE corrected, cluster size = 309). Greater activation was observed for pseudowords in the left caudate, putamen, precentral gyrus, inferior frontal gyrus, middle frontal gyrus, inferior parietal lobule, precuneus, and supramarginal gyrus (see Fig 4 and Table 3). This pattern of increased activation for novel relative to existing words has previously been observed in several neuroimaging studies [114, 115].

**Table 3:** Neural activation differences by condition and group.

Region	BA	Volume	X	Y	Z	Peak Activation	p value
**Pseudoword > Word**							
**L. Caudate, L. Putamen**		363	-10.5	4.5	17.5	0.416	<.02
**L. Precental Gyrus, L. Inferior Frontal Gyrus, L. Middle Frontal Gyrus**	6/9/43/44	355	-46.5	1.5	29.5	0.395	<.03
**L. Inf Parietal Lobule, L. Precuneus, L. Supramarginal Gyrus**	39/40	325	-40.5	-43.5	35.3	0.316	<.04
**Val/Met > Val/Val**							
**L. R. Precuneus, L. Inf Parietal Lobule**	7/31/40	1305	-10.5	-70.5	32.5	0.3105	< <.01
**L. R. Hippocampus, L. R. Parahippocampal Gyrus, L. R. Fusiform Gyrus, Cerebellum**	19/37	1063	-25.5	-55.5	-6.5	0.3935	< <.01
**L. Mid Frontal Gyrus, L. Inf Frontal Gyrus, L. Thalamus**	9/34	689	-34.5	-16.5	-18.5	0.2733	<.01
**R. Cingulate, R. Mid Frontal Gyrus, R. Sup Frontal Gyrus**	6/8/32	609	28.5	4.5	47.5	0.3051	<.01
**L. Cingulate, L. Medial Frontal Gyrus, L. Mid Frontal Gyrus, L. Precental Gyrus**	4/6/24/31	464	-19.5	-7.5	50.5	0.2803	<.01
**R. Sup Temporal Gyrus, R. Inf Parietal Lobule, R. Sup Parietal Lobule**	7/22/39/40	384	49.5	-43.5	17.5	0.3445	<.02

For all regions showing significant differences in neural activation, Brodmann Area (BA), Cluster Volume (in voxel number), MNI coordinates at peak, maximum peak activation, and p-value for peak activation. The sign of the maximum peak activation indicates the directionality of the observed effect.

#### Genotype: Val/Val homozygotes vs. Met allele carriers

**Fig 5:**
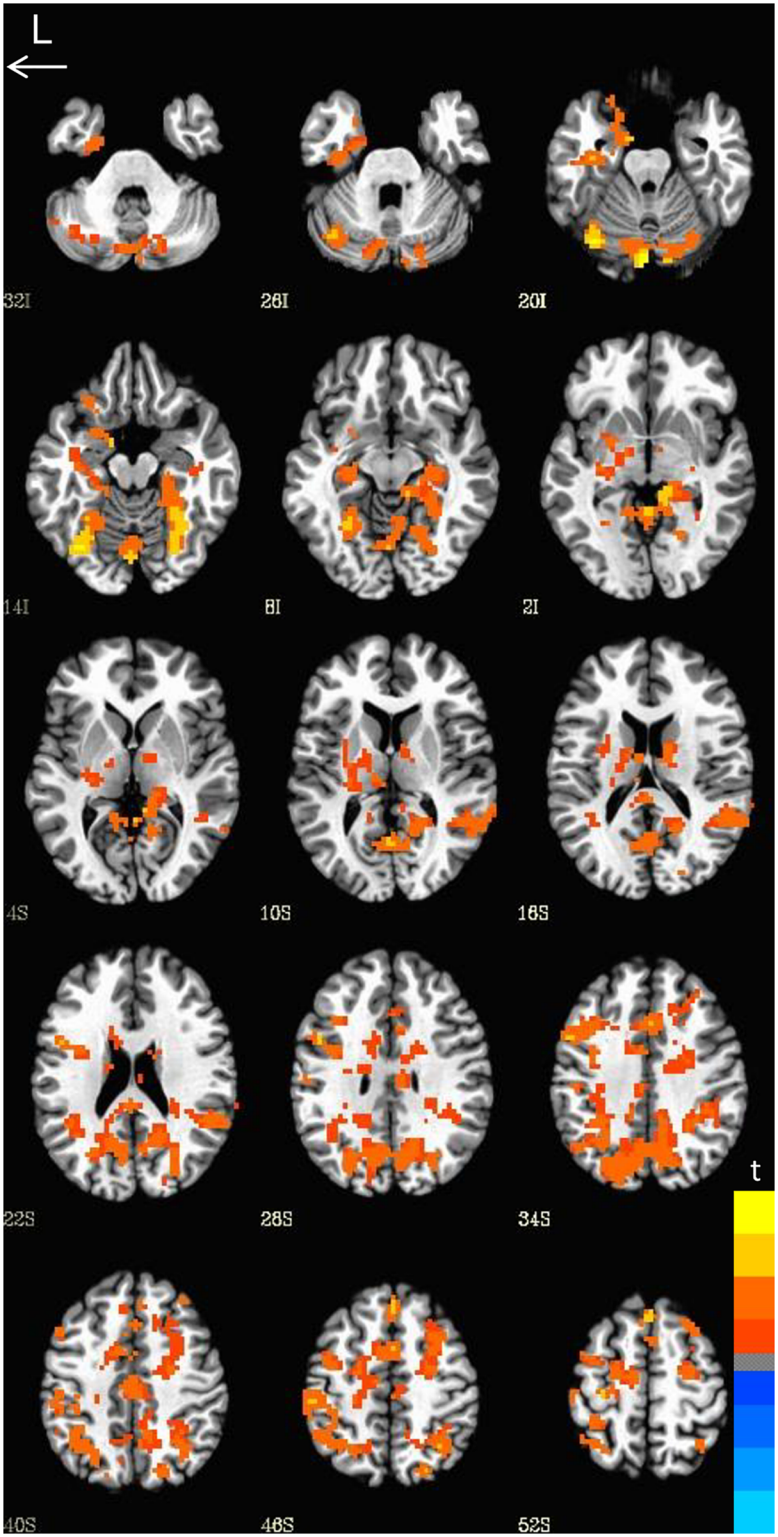
Neural activation by group. Comparisons of the Val/Val homozygotes and Met carriers. The Met carriers showed greater activation relative to the Val/Val homozygotes.

We observed a significant main effect of genotype. Comparisons of the two genetic groups revealed several regions of greater activation for Met allele carriers relative to Val/Val homozygotes (*t* = 1.993, *p* =.05, FWE corrected, cluster size = 309). We did not observe significant interactions between genotype and word type, therefore, our analysis was focused on patterns of neural activation for both words and pseudowords combined as an index of print decoding. Met allele carriers showed greater activation relative to Val/Val homozygotes in the bilateral hippocampus, bilateral parahippocampal gyrus, bilateral fusiform gyrus, bilateral cingulate, bilateral precuneus, bilateral inferior parietal lobule, and bilateral middle frontal gyrus, left inferior frontal gyrus, left medial frontal gyrus, left precentral gyrus, and left thalamus, and right superior parietal lobule, right superior frontal gyrus, and right superior temporal gyrus (see Fig 5 and Table 3). The left inferior frontal gyrus, left inferior parietal lobule (IPL), left cuneus/precuneus, and the left fusiform gyrus (which includes the region referred to as the visual word form area) are areas crucially involved in aspects of reading [80, 81, 87, 100, 101, 116]. Moreover the thalamus and putamen have been recently implicated in reading and associated with “late talking” [89, 90]. The hippocampus and parahippocampal regions, have been previously associated with learning and memory [7, 9, 15, 117].

There were no areas where the Val/Val homozygotes showed greater activation relative to Met allele carriers. Table 3 shows a summary of all regions of activation.

#### Brain Behavior Correlations

**Fig 6:**
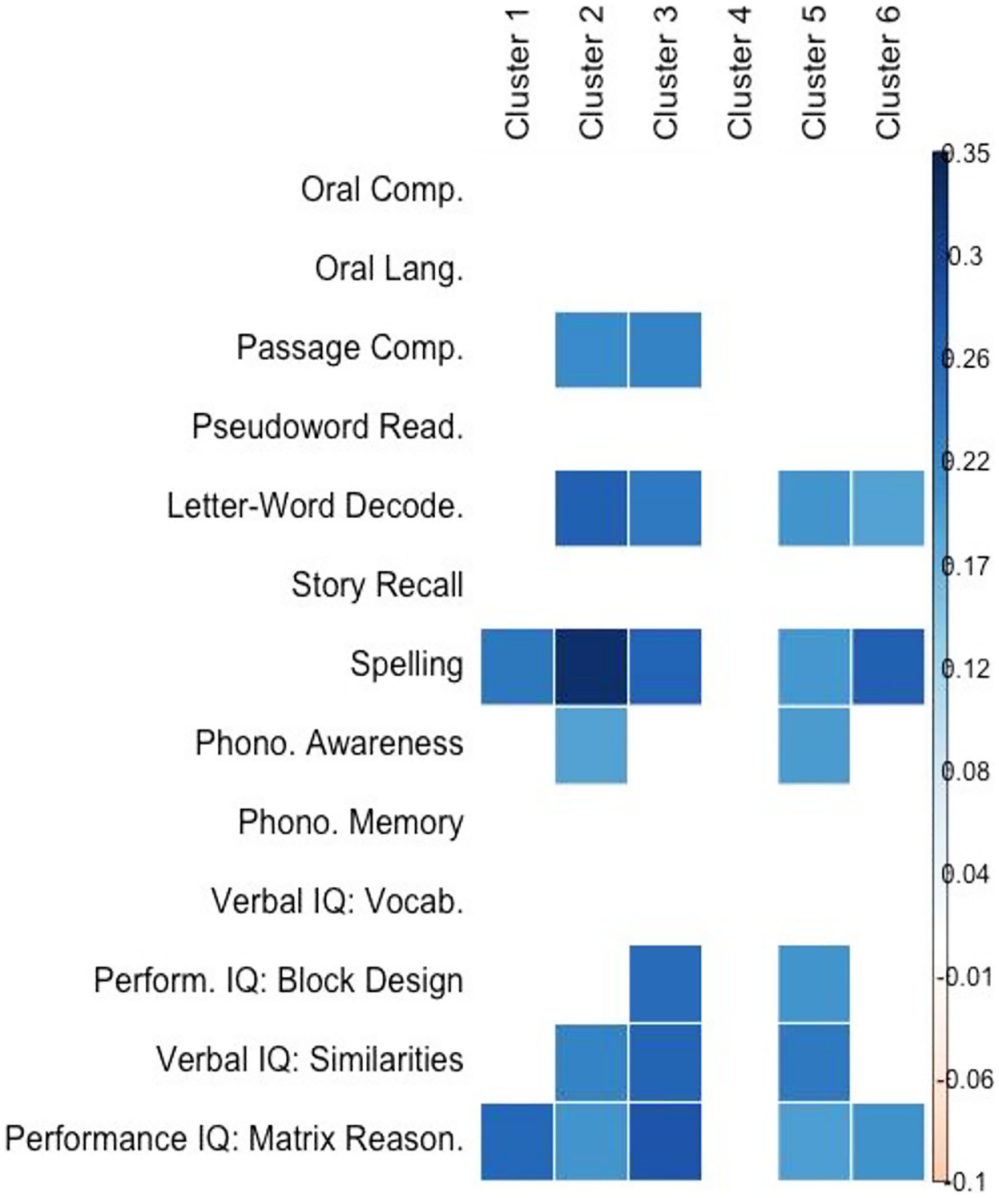
Brain-behavior correlations. Correlations between children’s performance on behavioral battery and mean activation in regions reported in Table 3 (regions where Met allele carriers showed greater activation than Val/Val homozygotes). All correlations are significant at p <.05, after permutation testing.

Across groups, neural activation in these regions were significantly correlated (p <.05) with children’s performance on Passage Comprehension, Letter-Word Decoding, Spelling, Phonological Awareness, and Verbal (Similarities and Vocabulary) and Performance (Block Design and Matrix Reasoning) IQ measures (see Fig 6). Skills identified in this analysis include some that significantly differentiated our groups, as well as other skills that did not, but that are correlated with those group- associated skills.

#### Age

Our analyses also revealed a significant effect of age on patterns of neural activation.

Increased age was associated with greater activation in the right cuneus and the left middle frontal gyrus for both groups. There were no significant interactions between age and genotype, and no significant effects of other covariates.

## Discussion

The central research questions of the present study was whether a common genetic variant in the *BDNF* gene (the Val^66^Met polymorphism) modulates patterns of neural activation in the developing brain in ways that are relevant for children’s reading skills. We approached this research question using a combined “genes-brain-behavior” method with the aim to unravel new information about the biological underpinnings of the development of reading and reading-related skills. Indeed, we observed differences between children who had at least one Met allelle (Val/Met or Met/Met) and those who were homozygous for the Val allele (Val/Val) in behavioral performance on tasks tapping memory and in patterns of functional neural activation during reading.

Children who were Val/Val homozygotes outperformed Met allele carriers on measures of phonological memory, reading comprehension and nonverbal IQ (block design subtest). These findings are consistent with the previously documented association in adults between the Val^66^Met polymorphism in the *BDNF* gene and memory and cognitive function [15–21].

We did not observe significant group differences on any of our other language or reading measures, though we did see an overall trend in the data for higher performance among Val/Val homozygotes, which is consistent with the high intercorrelations among these skills. These findings are consitent with our predictions that *BDNF* may be associated with reading ability through an effect on memory-related skills that are important for reading. Specifically, at the level of phonology, the capacity to monitor and maintain sounds in a phonological loop system via phonological working memory [62] contributes to a child’s phonological awareness skills, which are known precursors to reading development. At the level of text processing, a reader must attend to and maintain incoming information in memory as they accumulate knowledge across phrases and sentences [118, 119]. Indeed, many children who show reading impairments (at the level of the word and at the level of text) show working memory deficits [119–122].

Our primary fMRI analysis revealed greater neural activation during reading in children who were Met allele carriers compared with Val/Val homozygotes. The areas where we observed greater neural activation in Met allele carriers relative to Val/Val homozygotes included a broad network of regions known to be important for reading in children, including left (and right) fusifrom gyrus, left inferior frontal gyrus, and left superior temporal gyrus. Specifically, the left fusiform gyrus is strongly associated with visual word processing, the left inferior frontal gyrus, has been implicated in lexical, morphological and syntactic processing, and the left superior temporal gyrus, is involved in spoken language and phonological processing. Increased activation in left hemisphere reading and language regions is frequently associated with increased task difficulty, particularly during reading [99, 101, 123]. There were no areas that showed greater activation for Val/Val homozygotes compared to Met allele carriers.

We also observed differences in functional activation as a function of genotype in the hippocampus and parahippocampal gyrus. These findings are consistent the known function of BDNF in regulating hippocampal and parahippocampal function and volume [9, 12, 36, 45, 124]. Differences in hippocampal activity during recognition of words and pseudowords also aligns with findings suggesting that learning words (like many other forms of learning) involves complementary learning systems supported in part by the hippocampus [125]. Specifically, this account suggests that newly learned words are first represented in the hippocampal system and are then slowly integrated in to the cortex over a period of time that includes offline sleep. Studies have also shown advantages (e.g., faster retrieval) for words that have been consolidated after a period of offline sleep, and also reduced hippocampal activity during retrieval of consolidated words [125–128]. On possible implication of our findings is that Val/Val homozygotes (who show less hippocampal activation during reading) may have an advantage in learning and consolidation that contributes to improved reading-related skills, compared to Met allele carriers, for whom reading may be more effortful.

Our findings support emerging cumulative risk models of reading development that suggest that multiple cognitive and linguistic components give rise to skilled reading in the course of normal development and can also constitute vulnerabilities of the reading system. Children who carry the Met allele at rs6562 of the *BDNF* gene, showed overall lower scores on measures of phonological working memory and reading comprehension, and in functional neural activation in regions that support reading. Our findings suggest that, development of word reading proficiency may be facilitated by compensatory neural resources in individuals who have a genetic predisposition for poorer memory performance, including increased activation in classic language areas and in regions that support learning and memory more generally (i.e. the hippocampus and parahippocampal gyrus). This conclusion is also consistent with findings that training on working memory tasks has positive transfer effects in reading comprehension [129, 130].

It is important to note that this study is not without limitations. While we have observed brain and behavioral differences between children who were Val/Val homozygotes and Met allele carriers, we have only investigated a single nucleotide polymorphism within one gene. Our ongoing work includes genome-wide association approaches, which allow for the investigation of more genes and relationships among genes. Our sample size of 81 children, while considerable for combined gene- brain- behavior approaches is still modest relative to large scale association studies. Further, our age range limits stronger conclusions about developmental trajectories for the influence of the *BDNF* Val^66^Met polymorphism.

This first-time investigation of the role of the *BDNF* Val^66^Met polymorphism in functional activation underlying reading in the children suggests that homozygous Val carriers might have a cognitive advantage over Met allele carriers with respect to the brain’s capacity to learn to read and that neural markers typically associated reading skill are associated with the *BDNF* Val^66^Met polymorphism. While the current study has a modest sample size, our combined “genes-brain-behavior” approach in the study of a common genetic variant contributes to the growing literature on the neurogenetic foundations of reading development.

Future work will explore whether the relationships identified here can be used to enhance the prediction of the onset of reading difficulty and refine our understanding of the nature of reading disability.

## Appendix

### Participant Race and Ethnicity

With respect to race and ethnicity, the vast majority of the participants in both groups (N = 70) were Caucasian (49 Val/Val and 21 Met allele carriers). Of the remaining eleven participants, one participant was of African-American ethnicity, two participants were of Hispanic ethnicity, three participants were of Asian ethnicity, and five participants of mixed ethnicity. The *BDNF* rs6265 SNP can have minor allele frequencies (MAF) that differ based on ethnicity. For example: *BDNF* rs6265 MAF is about 20% for Caucasian, 4% for African American, and 40% for Asian population [131]. Our study had a small number of participants who were not of Caucasian ethnicity, and preclude analysis of differences among ethnic groups. Future work with larger sample sizes will necessitate comprehensive exploration of the variation between ethnic groups based on minor allele frequencies.

### Participant Stress

In order to assess stress, we collected information about stressful events in each child’s life. This included any changes to family structure (birth/adoption of new child, parent divorce, remarriage of parent, absence of parent), changes in location (new school, moving to new city), death and/or illness in the family, and custody (parent voluntarily gave up custody, parent found unfit by court system). On average, the children in our sample experience 1.5 stressful events, and there were no significant differences between groups (M_Val/Val_ = 1.62, SD = 2.80; M_Met carrier_ = 1.15, SD = 1.54, *F*(79) = 0.626, *p*>.05).

## Supporting Information

S1 Dataset Statistical fMRI maps in AFNI format for each subject. (ZIP)

S1 File Analysis script in AFNI (Analysis of Functional Neuroimages software package) used to analyze data. (SH)

S1 Table List of behavioral assessments. Each behavioral measure from the corresponding assessment battery is listed. Mean and by-age reliability coefficients for each measure are included.
(TIFF)
