# Supplementary figures and images for: The BDNF Val66Met Polymorphism Influences Reading Ability and Patterns of Neural Activation in Children
Source: PLoS One. 2016 Aug 23;11(8):e0157449. doi: 10.1371/journal.pone.0157449 (PMC4995017; doi:10.1371/journal.pone.0157449)

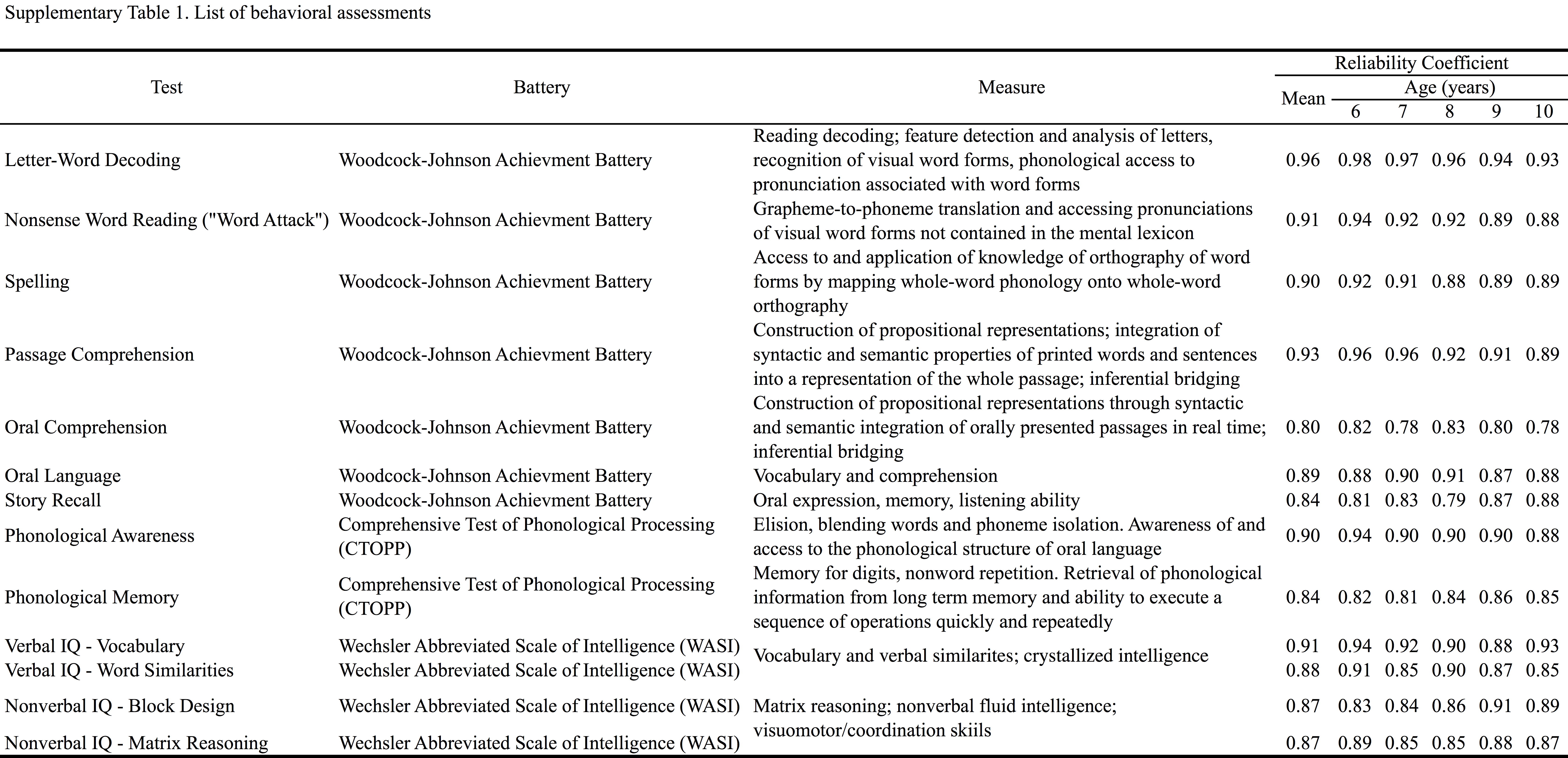

Supplement: S1 Table — Each behavioral measure from the corresponding assessment battery is listed. Mean and by-age reliability coefficients for each measure are included. (TIFF) [file pone.0157449.s003.tiff]
